# Designing combinational herbal drugs based on target space analysis

**DOI:** 10.1186/s12906-024-04455-9

**Published:** 2024-05-01

**Authors:** Assefa Mussa Woyessa, Lemessa Etana Bultum, Doheon Lee

**Affiliations:** 1 Department of Bio and Brain Engineering, Korea Advanced Institute of Science and Technology, Daejeon, 34141 South Korea; 2 Bio-Synergy Research Center, Daejeon, 34141 South Korea; 3 Institute of Agricultural Life Sciences, Dong-A University, Busan, 49315 South Korea

**Keywords:** Herbal medicines, Holistic approach, Target space analysis, Traditional oriental medicines

## Abstract

**Background:**

Traditional oriental medicines (TOMs) are a medical practice that follows different philosophies to pharmaceutical drugs and they have been in use for many years in different parts of the world. In this study, by integrating TOM formula and pharmaceutical drugs, we performed target space analysis between TOM formula target space and small-molecule drug target space. To do so, we manually curated 46 TOM formulas that are known to treat Anxiety, Diabetes mellitus, Epilepsy, Hypertension, Obesity, and Schizophrenia. Then, we employed Absorption, Distribution, Metabolism, Excretion, and Toxicity (ADMET) properties such as human ether-a-go-go related gene (hERG) inhibition, Carcinogenicity, and AMES toxicity to filter out potentially toxic herbal ingredients. The target space analysis was performed between TOM formula and small-molecule drugs: (i) both are known to treat the same disease, and (ii) each known to treat different diseases. Statistical significance of the overlapped target space between the TOM formula and small-molecule drugs was measured using support value. Support value distribution from randomly selected target space was calculated to validate the result. Furthermore, the Si-Wu-Tang (SWT) formula and published literature were also used to evaluate our results.

**Result:**

This study tried to provide scientific evidence about the effectiveness of the TOM formula to treat the main indication with side effects that could come from the use of small-molecule drugs. The target space analysis between TOM formula and small-molecule drugs in which both are known to treat the same disease shows that many targets overlapped between the two medications with a support value of 0.84 and weighted average support of 0.72 for a TOM formula known to treat Epilepsy. Furthermore, support value distribution from randomly selected target spaces in this analysis showed that the number of overlapped targets is much higher between TOM formula and small-molecule drugs that are known to treat the same disease than in randomly selected target spaces. Moreover, scientific literature was also used to evaluate the medicinal efficacy of individual herbs.

**Conclusion:**

This study provides an evidence to the effectiveness of a TOM formula to treat the main indication as well as side effects associated with the use of pharmaceutical drugs, as demonstrated through target space analysis.

**Supplementary Information:**

The online version contains supplementary material available at 10.1186/s12906-024-04455-9.

## Background

Traditional oriental medicines (TOMs) have been in use by many people around the world for many years. Traditional Chinese medicine (TCM), African traditional medicine, and Kampo medicine provide a wide variety of data on medicinal plants [1–4]. Furthermore, TCM has been widely used as a primary source of therapy in China for many years [4]. TOMs, unlike pharmaceutical drugs, follow a distinct diagnostic methodology since they view disease as a common product of both maladjustment in the human body and pathogenetic factors [4, 5]. Most pharmaceutical drugs, also called western medicine, are designed with a single active component or ingredient that targets a specific ion channel, receptor, regulatory protein, or enzyme that caused a disease [5, 6]. Considering how complex a human body is, multi-compound multi-target approaches are getting more interest as a potential treatment, particularly for complex diseases. Different researchers have diverse opinions concerning the therapeutic efficacy and mechanism of herbal medicine [7–9]. Even though we do not fully understand the mechanisms of most herbal medicines, they have been used as a primary source of medication and as a supplement to pharmaceutical drugs in both developing and developed countries [10, 11]. Studies have shown that around 20% of the US population as well as around 23% of preoperative and ambulatory patients used herbal medicine [12–14]. Strohl (2000) showed that more than one-third of most marketed drugs are natural product-derived ones [15]. Despite several studies being carried out to investigate the efficacy of TOM-derived compounds, their mechanisms of action and therapeutic efficacy are not well known compared to pharmaceutical drugs [15–17]. Despite the lack of clear understanding regarding the underlying mechanisms of action, few studies have reported the presence of herbal synergism [18–21]. Furthermore, due to the multi-compound nature of traditional medicine and natural products, the molecular mechanisms of traditional medicine still stay largely unknown. Due to their wide use as well as their important role in the drug discovery process, the need and interest to investigate the mechanisms of TOMs increased rigorously.

**Fig. 1:**
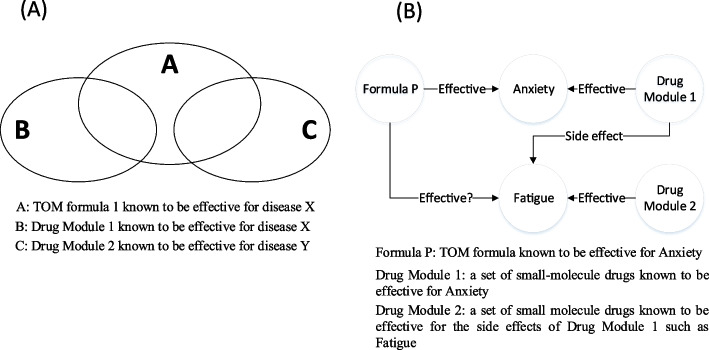
Concept of the target space analysis. Three target spaces – TOM formula 1 target space, Drug Module 1 target space, and Drug Module 2 target space – were analyzed. Figure (**A**): TOM formula 1 and Drug Module 1 are known to treat disease X whereas Drug Module 2 are known to treat the side effects of Drug Module 1 i.e. disease Y. The target space analysis aims to provide scientific evidence about the effectiveness of TOM formula 1 to treat the main indication (disease X) with side effects (disease Y). Figure (**B**): Example of target space analysis between TOM Formula P, which is known to be effective for Anxiety, Drug Module 1 is a set of small-molecule drugs that are known to treat Anxiety, and Drug Module 2 is a set of small-molecule drugs that are known to treat the side effects of Drug Module 1 such as Fatigue. The purpose of the target space analysis is to investigate if the target space of TOM formula P is significantly overlapped against the target space of both Drug Module 1 and Drug Module 2. In addition, this study evaluates If the overlapped targets are significant and higher than the randomly expected target space overlap

**Fig. 2:**
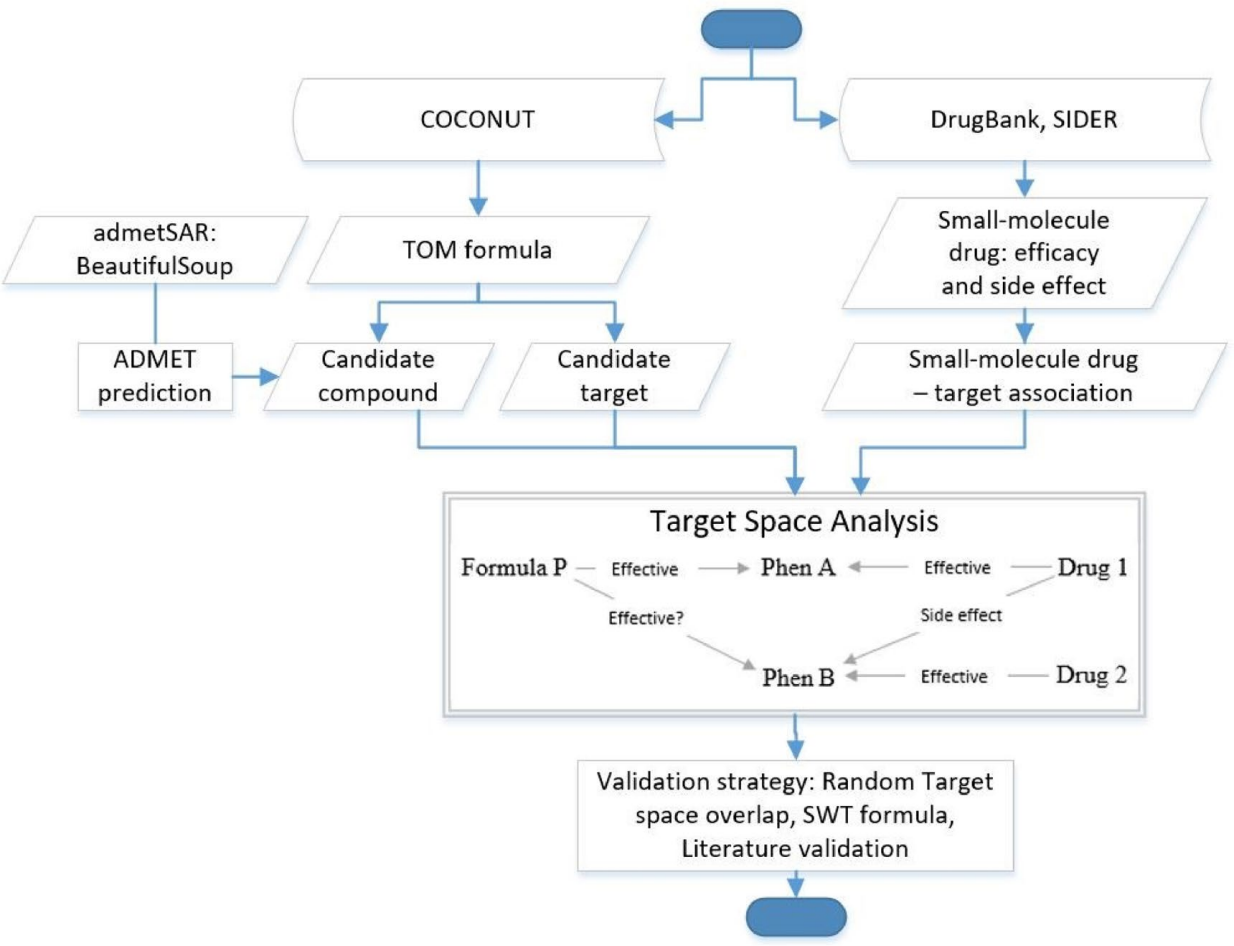
Overall workflow and scheme of this study. First, Traditional oriental medicine (TOM) and small-molecule drug-related data were curated from COCONUT and DrugBank, respectively. Second, target space analysis was performed between the TOM formula and small-molecule drug target spaces. By using target space analysis, this study aims to provide scientific evidence if TOM formula P can be used for both the main indication Phenotype A and the side effects of Drug Module 1 which is Phenotype B (both Drug Module 1 and Formula P are known to treat Phenotype A). We performed target space analysis between Formula P against Drug Module 1, and Formula P against Drug Module 2 target spaces. We validate our result using randomly selected target space analysis, SWT formula, and published literature

In this study, we perform target space analysis to provide scientific evidence about the efficacy of traditional oriental medicine formulas. The target space analysis focuses on the target spaces of two different treatments, TOM formula, and small-molecule drugs, both known to treat the same disease. Furthermore, with the multi-component multi-target principle of the TOM formula [5], we investigate the effectiveness of herbal medicine formulas in treating more indications in addition to the main one it is designed to treat (see Fig. 1). In the present study, six phenotypes – Anxiety, Diabetes mellitus, Epilepsy, Hypertension, Obesity, and Schizophrenia – were selected from three different disease ontologies, cardiovascular system disease, metabolic system disease, and neurological disorder. To validate our result, we perform target spaces analysis between randomly selected target space of TOM formula and small-molecule drugs. In addition, the SWT formula and published literature were used to validate our results. The flowchart in Fig. 2 shows the overall process of the present study (see Materials and methods section).

## Results

### TOM formula versus small-molecule drugs: both medicines are known to be effective for the same disease

The target space analysis showed that a significant number of targets overlapped between the TOM formula and Drug Module 1. The number of target genes associated with TOM formula and Drug Module 1, in which both medicines are known to treat the same disease, were significant (see Additional file 1). In the present study, Cytochrome P450 (CYP) isozymes were excluded from the target space analysis since their primary role is in the metabolism of drugs and other xenobiotics [22]. For instance, the result of the target space analysis between TOM formula 1, TOM formula 2, TOM formula 3, TOM formula 4, TOM formula 5, TOM formula 6, TOM formula 7, TOM formula 8, TOM formula 9, and TOM formula 10 against small-molecule drugs in which both medications are known to treat Anxiety showed that the two medicines are associated with 17, 16, 18, 18, 22, 7, 16, 24, 19, and 25 target genes, respectively. The 17 targets which are overlapped between TOM formula 1 and small-molecule drugs are ALB, GABRB1, GABRB2, GABRB3, GABRD, GABRG1, GABRG2, GABRP, GABRR1, GABRR2, HTR1A, ORM1, PTGS1, TSPO, UGT1A9, UGT2B15, and UGT2B7. Similarly, the target space analysis between TOM formula 1, TOM formula 2, TOM formula 3, TOM formula 4, TOM formula 5, TOM formula 6, TOM formula 7, TOM formula 8, TOM formula 9, and TOM formula 10 against small-molecule drugs in which both medications are known to treat Diabetes mellitus showed that the two medicines are associated with 55, 20, 62, 6, 60, 63, 14, 37, 47, and 65 target genes, respectively. Furthermore, the results of most of the target space analyses performed in this study showed that a significant number of targets overlapped between TOM formula target space and small-molecule drug target space.

Statistical significance of the overlapped targets between a TOM formula and small-molecule drug target spaces were calculated using support, overlapping target space divided by pharmaceutical drugs target space. Then, the capability of a single TOM formula to treat both the main indication and other indications (side effects) is measured as the summation of individual support values divided by the total number of indications (Tables 1 and 2).

**Table 1:** Target Space analysis between TOM formula used for Anxiety and small-molecule drug

	F1	F2	F3	F4	F5	F6	F7	F8	F9	F10
Drugs (Anxiety)	0.63	0.59	0.67	0.67	0.81	0.26	0.59	0.89	0.70	0.91
Drugs (Fatigue)	0.44	0.44	0.44	0.44	0.44	0.22	0.44	0.44	0.44	0.44
Drugs (Hypotension)	0.66	0.70	0.70	0.62	0.70	0.13	0.70	0.66	0.75	0.66
Drugs (Somnolence)	0.5	0.5	0.5	0.5	0.5	0.31	0.5	0.5	0.5	0.5
Drugs (Constipation)	0.69	0.69	0.66	0.60	0.67	0.29	0.66	0.64	0.69	0.67
Support (weighted avg.)	0.60	0.59	0.62	0.61	0.69	0.25	0.58	0.73	0.65	0.74

Statistical significance of targets overlapped between TOM formulas (F1, F2… F10) used to treat Anxiety, pharmaceutical drugs (Drug Module 1) used to treat Anxiety, and pharmaceutical drugs (Drug Module 2) known to treat side effects of Drug Module 1 – Fatigue, Hypotension, Somnolence, and Constipation. The support value showed that the majority of TOM formulas were significantly associated with targets of both Drug Module 1, small-molecule drugs known to be effective for Anxiety – the main indication, and Drug Module 2, small-molecule drugs known to be effective for Fatigue, Hypotension, Somnolence, and Constipation – side effects of Drug Module 1

**Table 2:** Target Space analysis between TOM formula used for Epilepsy and small-molecule drug

	F1	F2	F3	F4	F5	F6	F7
Drugs (Epilepsy)	0.84	0.78	0.78	0.61	0.65	0.32	0.78
Drugs (Somnolence)	0.44	0.44	0.50	0.44	0.50	0.31	0.50
Drugs (Fatigue)	0.43	0.41	0.43	0.38	0.43	0.22	0.43
Drugs (Skin rash)	0.70	0.70	0.70	0.70	0.70	0.25	0.70
Drugs (Nausea)	0.72	0.66	0.70	0.51	0.53	0.13	0.68
Drugs (Insomnia)	0.69	0.66	0.66	0.49	0.52	0.16	0.65
Drugs (Tremor)	0.62	0.54	0.58	0.46	0.50	0.17	0.58
Support (weighted avg.)	0.72	0.68	0.69	0.55	0.59	0.26	0.69

Statistical significance of targets overlapped between TOM formulas (F1, F2… F7) used to treat Epilepsy, pharmaceutical drugs (Drug Module 1) used to treat Epilepsy, and pharmaceutical drugs (Drug Module 2) known to treat side effects of Drug Module 1 – Somnolence, Fatigue, Skin rash, Nausea, Insomnia, and Tremor. The support value showed that the majority of TOM formulas were significantly associated with the targets of both Drug Module 1 which are small-molecule drugs known to be effective for the main indication i.e. Epilepsy, and Drug Module 2 which are small-molecule drugs known to treat the side effects of Drug Module 1 i.e. Somnolence, Fatigue, Skin rash, Nausea, Insomnia, and Tremor

### TOM formula versus small-molecule drugs: each medicine is known to be effective for different disease

The previous section showed the presence of a significant target space overlap between a TOM formula and small-molecule drugs in which both medicines are known to treat the same disease. However, as several studies reported, small-molecule drugs have undesired effects – adverse side effects. In this section, we analyzed the target spaces of the TOM formula which are known to treat phenotype A, small-molecule drugs (Drug Module 1) which are known to treat phenotype A, and small-molecule drugs (Drug Module 2) which are known to treat the side effects of Drug Module 1. The objective is to find out if a single TOM formula is significantly associated with the targets of both Drug Module 1 and Drug Module 2 (see Fig. 2). If there is significant target space overlap, then we might say a TOM formula has the potential to treat phenotype A and alleviate possible side effects that might come from Drug Module 1.

**Fig. 3:**
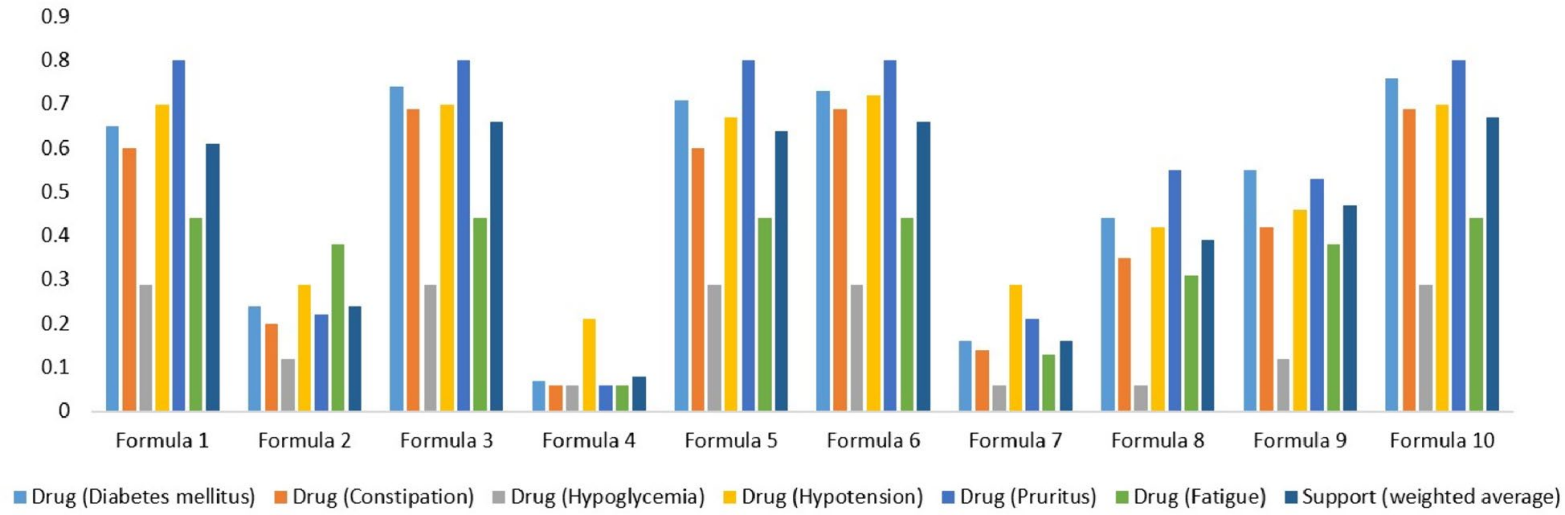
Statistical significance of targets overlapped between TOM formulas (Formula 1, Formula 2… Formula 10) used to treat Diabetes mellitus, pharmaceutical drugs (Drug Module 1) used to treat Diabetes mellitus, and pharmaceutical drugs (Drug Module 2) known to treat side effects of Drug Module 1 – Constipation, Hypoglycemia, Hypotension, Pruritus, and Fatigue. The support value showed that the majority of TOM formulas were significantly associated with targets of both Drug Module 1, small-molecule drugs known to be effective for Diabetes mellitus – a main indication, and Drug Module 2, small-molecule drugs known to be effective for Constipation, Hypoglycemia, Hypotension, Pruritus, and Fatigue – side effects of Drug Module 1

**Fig. 4:**
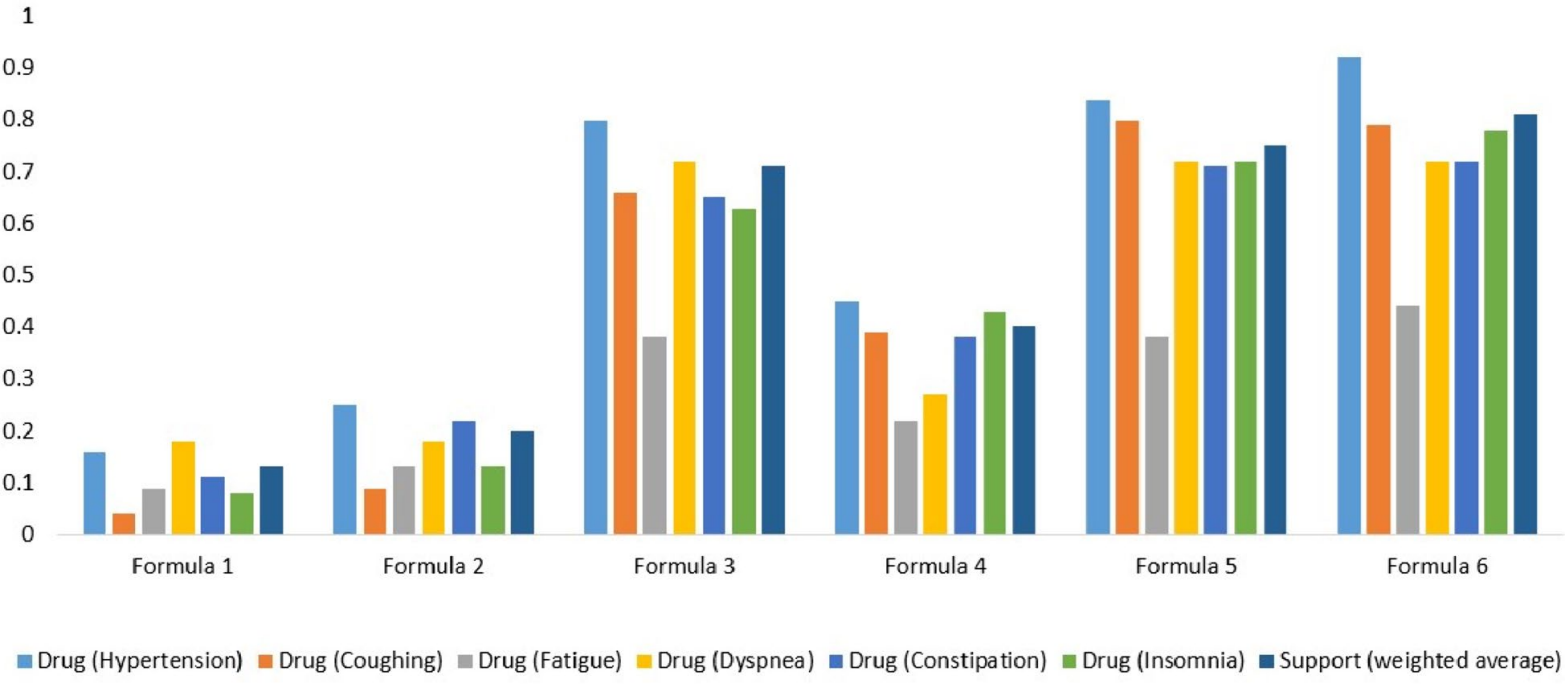
Statistical significance of targets overlapped between TOM formulas (Formula 1, Formula 2… Formula 6) used to treat Hypertension, pharmaceutical drugs (Drug Module 1) used to treat Hypertension, and pharmaceutical drugs (Drug Module 2) known to treat side effects of Drug Module 1 – Coughing, Fatigue, Dyspnea, Constipation, and Insomnia. The support value showed that the majority of TOM formulas were significantly associated with targets of both Drug Module 1, small-molecule drugs known to be effective for Hypertension – a main indication, and Drug Module 2, small-molecule drugs known to be effective for Coughing, Fatigue, Dyspnea, Constipation, and Insomnia – side effects of Drug Module 1

**Fig. 5:**
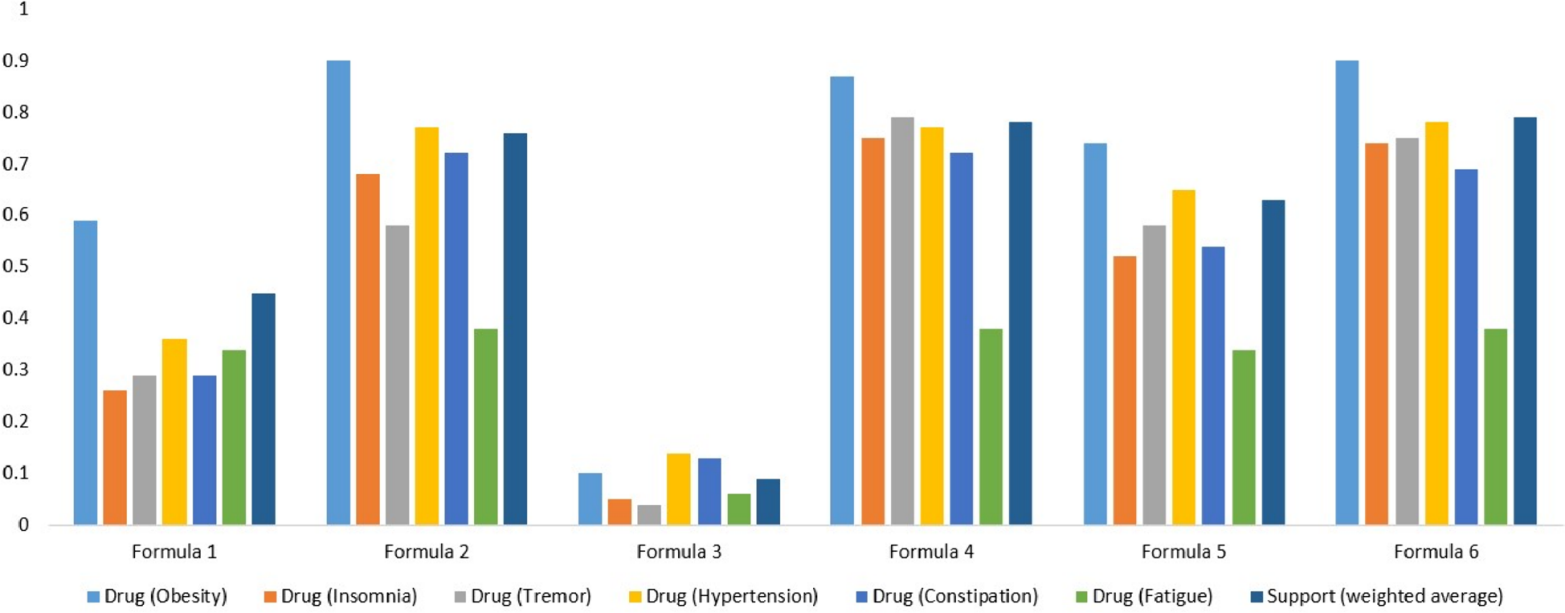
Statistical significance of targets overlapped between TOM formulas (Formula 1, Formula 2… Formula 6) used to treat Obesity, pharmaceutical drugs (Drug Module 1) used to treat Obesity, and pharmaceutical drugs (Drug Module 2) known to treat side effects of Drug Module 1 – Insomnia, Tremor, Hypertension, Constipation, and Fatigue. The support value showed that the majority of TOM formulas were significantly associated with targets of both Drug Module 1, small-molecule drugs known to be effective for Obesity – a main indication, and Drug Module 2, small-molecule drugs known to be effective for Insomnia, Tremor, Hypertension, Constipation, and Fatigue – side effects of Drug Module 1

**Fig. 6:**
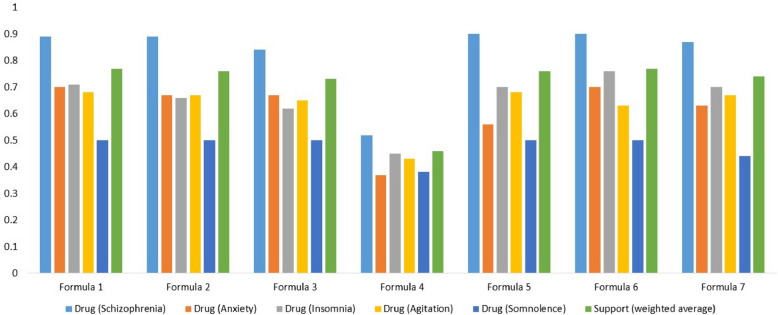
Statistical significance of targets overlapped between TOM formulas (Formula 1, Formula 2… Formula 7) used to treat Schizophrenia, pharmaceutical drugs (Drug Module 1) used to treat Schizophrenia, and pharmaceutical drugs (Drug Module 2) known to treat side effects of Drug Module 1 – Anxiety, Insomnia, Agitation, and Somnolence. The support value showed that the majority of TOM formulas were significantly associated with targets of both Drug Module 1, small-molecule drugs known to be effective for Schizophrenia – a main indication, and Drug Module 2, small-molecule drugs known to be effective for Anxiety, Insomnia, Agitation, and Somnolence – side effects of Drug Module 1

Some of the side effects of the small-molecule drugs known to treat Anxiety are Fatigue, Hypotension, Somnolence, and Constipation [23–26]. We manually curated targets of the small-molecule drugs known to treat these side effects from DrugBank and then we build a target space. Then, we perform target space analysis between the TOM formula known to treat Anxiety (Formula 1, Phen A) and small-molecule drugs used to treat these side effects – Fatigue, Hypotension, Somnolence, and Constipation (Drug Module 2, Phen B). The number of targets overlapped between the two target spaces is summarized in Table 1. Similarly, some of the reported side effects of the small-molecule drugs known to treat Diabetes mellitus are Constipation, Hypoglycemia, Hypotension, Pruritus, and Fatigue [23, 24, 27, 28]. The small-molecule drugs used to treat these side effects as well as their targets were manually curated from DrugBank to build a target space. Then, we perform target space analysis between the TOM formula used to treat Diabetes mellitus (Formula1, Phen A) and small-molecule drugs used to treat these side effects – Constipation, Hypoglycemia, Hypotension, Pruritus, and Fatigue (Drug Module 2, Phen B) (see Fig. 3). Small-molecule drugs used for Epilepsy are reported to cause side effects such as Somnolence, Fatigue, Skin rash, Nausea, Insomnia, and Tremor [23, 24, 29]. Similarly, we curated the small-molecule drugs known to treat these side effects from DrugBank along with their targets and built a target space. Next, we perform target space analysis between the TOM formula used to treat Epilepsy (Formula 1, Phen A) and small-molecule drugs used to treat these side effects – Somnolence, Fatigue, Skin rash, Nausea, Insomnia, and Tremor (Drug Module 2, Phen B) (Table 2). Furthermore, small-molecule drugs known to treat Hypertension are reported to cause side effects such as Insomnia, Coughing, Fatigue, Constipation, and Dyspnea [23, 24, 30]. Small-molecule drugs known to treat these side effects along with their targets were manually curated from DrugBank and a target space was built. Then, we perform target space analysis between the TOM formula used to treat Hypertension (Formula 1, Phen A) and small-molecule drugs used to treat these side effects – Insomnia, Coughing, Fatigue, Constipation, and Dyspnea (Drug Module 2, Phen B) (see Fig. 4). Similarly, small-molecule drugs used for Obesity are known to cause side effects such as Insomnia, Tremor, Hypertension, Constipation, and Fatigue [23, 24, 31]. We curated small-molecule drugs known to treat these side effects together with their targets from DrugBank. Next, target space analysis was performed between the TOM formula used to treat Obesity (Formula 1, Phen A) and small-molecule drugs known to treat these side effects – Insomnia, Tremor, Hypertension, Constipation, and Fatigue (Drug Module 2, Phen B) (see Fig. 5). Finally, small-molecule drugs used for Schizophrenia cause side effects such as Anxiety, Insomnia, Agitation, and Somnolence [23, 24, 32]. Small-molecule drugs known to treat these side effects together with their targets were manually curated from DrugBank. Then, we perform target space analysis between the TOM formula used to treat Schizophrenia (Formula 1, Phen A) and small-molecule drugs known to treat these side effects – Anxiety, Insomnia, Agitation, and Somnolence (Drug Module 2, Phen B) (see Fig. 6).

## Discussion

The previous section showed that the probability of a TOM formula treating the main indication with potential side effects was significant. In this section, we discuss the results of support value distribution from randomly selected target space, SWT formula, and published literature, which are used as a validation strategy.

### Support value distribution of randomly selected target space

Randomly selected target space analysis, SWT formula, and published literature were used to validate our results. Support value distribution for the randomly selected target spaces was calculated using two case studies. First, we used the target spaces of three TOM formulas each for both Obesity and Diabetes mellitus phenotype, small-molecule drugs known to treat Obesity (Drug Module 1a), small-molecule drugs known to treat Diabetes mellitus (Drug Module 1b), small-molecule drugs known to treat Malaria (Drug Module 2a), and small-molecule drugs known to treat Tuberculosis TB (Drug Module 2b). Then, we performed target space analysis between the TOM formula known to treat Obesity, Drug Module 1a, and Drug Module 2a. Similarly, we performed target space analysis between the TOM formula known to treat Diabetes mellitus, Drug Module 1b, and Drug Module 2b. The present study hypothesizes that there will be a higher target space overlap between a TOM formula and Drug Module 1 compared to the number of targets overlapped between the TOM formula and Drug Module 2. Results of the randomly selected target space analysis showed that more targets were overlapped between the TOM formula and Drug Module 1a which both are known to treat Obesity. Similarly, higher target overlap was reported between TOM formula and Drug Module 1b in which both are known to treat Diabetes mellitus. Second, to validate the effectiveness of a given TOM formula, we measured the support value distribution of randomly selected target spaces between a given TOM formula – TOM Formula 1, TOM formula 2, …, TOM formula 10 – known to treat Diabetes mellitus (referred as F1, F2, …, F10 throughout this section) against randomly selected target genes, generated from DrugBank. Drug Module 1b contains 38 small-molecule drugs, 84 target genes, and 88 small-molecule drug-target associations. For the randomly selected target genes, we collected more than 3000 target genes from DrugBank and we designed the randomly selected target spaces with a similar number of target genes as in Drug Module 1b. Then, we generate 1,000 random target spaces from 3,000 genes curated from DrugBank, and each randomly selected target space was designed to contain 84 target genes, the same number of targets as Drug Module 1b have. Then we performed target space analysis between F1, F2, …, F10, and the randomly selected target genes. We calculated support values for the 1,000 random sets and then we find the mean and variance of 1,000 support values. For instance, we performed target space analysis between F1 and randomly selected target genes. The target space analysis was performed for 1,000 random sets in which each random set was designed to have the same number of target genes as Drug Module 1b and we recorded 1,000 support values as a result. Then, we calculated the mean and variance of 1,000 support values and compared the significance of overlapped targets between the TOM formula and randomly selected target genes against the number of targets overlapped between TOM formulas and Drug Module 1b. The result of the support value distribution of the target space analysis showed that more targets were overlapped between TOM Formulas and Drug Module 1b compared to the number of targets overlapped between the TOM Formulas and randomly selected target genes. In general, the results of the support value distribution of randomly selected target space analysis are consistent with the findings of the current study. Tables 3, 4 and 5 showed support values for the overlapped targets between small-molecule drugs from DrugBank and TOM formula target space.

**Table 3:** Overlapped targets of the drugs known to treat Obesity (Drug Module 1a) and drugs known to treat Malaria (Drug Module 2a) against the TOM formulas known to treat Obesity

	TOM Formula 1	TOM Formula 2	TOM Formula 3
Drug Module 1a (Obesity)	0.89	0.87	0.89
Drug Module 2a (Malaria)	0.34	0.34	0.32

**Table 4:** Overlapped targets of the drugs known to treat Diabetes mellitus (Drug Module 1b) and drugs known to treat Tuberculosis TB (Drug Module 2b) against the TOM formulas known to treat Diabetes mellitus

	TOM Formula 1	TOM Formula 2	TOM Formula 3
Drug Module 1b (Diabetes mellitus)	0.74	0.73	0.76
Drug Module 2b (Tuberculosis TB)	0.37	0.39	0.41

**Table 5:** Overlapped targets of the drugs known to treat Diabetes mellitus (Drug Module 1b) and randomly selected target space from DrugBank against the TOM formulas known to treat Diabetes mellitus

	F1	F2	F3	F4	F5	F6	F7	F8	F9	F10
Drug Module 1b (Support)	0.65	0.24	0.74	0.07	0.71	0.73	0.16	0.44	0.55	0.76
Randomly selected target genes (μ)	0.49	0.22	0.50	0.04	0.51	0.53	0.08	0.21	0.40	0.56
Randomly selected target genes (σ2)	0.017	0.008	0.012	0.002	0.021	0.03	0.002	0.007	0.003	0.02

Target space overlap analysis of the TOM formulas that are known to treat diabetes mellitus against the target space of small-molecule drugs that are known to treat Diabetes mellitus (Drug Module 1b) and randomly selected target genes. The first row showed support values of targets overlapped between TOM formulas and Drug Module 1b. The second and third rows showed mean and variance of 1,000 support values calculated between TOM formulas and randomly selected target genes

### Validation using the Si-Wu-Tang formula

Si-Wu-Tang (SWT) is a popular Traditional Chinese Medicine (TCM) formula, composed of four herbs, Radix Angelicae Sinensis (AS), Rhizoma Ligustici Chuanxiong (LC), Radix Paeoniae Alba (PA), and Radix Rehmanniae Praeparata (RP), commonly used to treat various women’s disease, such as climacteric syndrome, menstrual discomfort, and other estrogen-related diseases [33, 34]. In addition to literature validation, we used this popular TCM formula to evaluate the findings of the present study. Herbal ingredients and targets associated with AS, PA, LC, and RP were curated from COCONUT and TCM Database@Taiwan. For AS, we curated 44 herbal ingredients, 3490 targets, and 6397 herbal ingredient–target associations. Similarly, for PA, we curated 51 herbal ingredients, 2206 targets, and 3235 herbal ingredient–target associations. For RP, we curated 22 herbal ingredients, 2603 targets, and 3836 herbal ingredient–target associations. Lastly, for LC, 6 herbal ingredients, 100 targets, and 105 herbal ingredient–target associations were curated (see Additional file 2).

Small-molecule drugs (Drug Module 1) that are known to treat women’s diseases, such as menstrual cramps, menopause, and climacteric syndrome, were curated from DrugBank. Therefore, we collected 31 small-molecule drugs and their respected targets (121 targets) to build Drug Module 1–target associations (see Additional file 3). Then, we performed target space analysis between Drug Module 1 target space and SWT target space. The analysis showed that 85 targets overlapped between Radix Paeoniae Alba (PA) and Drug Module 1, 74 targets overlapped between Radix Angelicae Sinensis (AS) and Drug Module 1, 51 targets overlapped between Radix Rehmanniae Praeparata (RP) and Drug Module 1, and 15 targets overlapped between Rhizoma Ligustici Chuanxiong (LC) and Drug Module 1. This shows that a significant number of targets overlapped between the SWT formula target space and Drug Module 1 target space. Support was used to measure the significance of the overlapped target space and the average support of the overlapped target space between the SWT formula and Drug Module 1 was 0.82.

According to SIDER and published literature, small-molecule drugs that are used to treat women’s disease (Drug Module 1) were reported to cause side effects such as pruritus, fatigue, nausea, hypotension, somnolence, insomnia, constipation, migraine, haemorrhage, and vulvovaginitis. Small-molecule drugs (Drug Module 2) that are known to treat these side effects were curated from DrugBank. We found eleven small-molecule drugs for pruritus, four small-molecule drugs for fatigue, thirteen small-molecule drugs for nausea, four small-molecule drugs for hypotension, two small-molecule drugs for somnolence, twenty-nine small-molecule drugs for insomnia, twenty-one small-molecule drugs for constipation, four small-molecule drugs for migraine, two small-molecule drugs for hemorrhage, and two small-molecule drugs for vulvovaginitis (see Additional file 4). Then, we perform target space analysis between Drug Module 2 and SWT formula target spaces and the statistical support of overlapped targets was 0.6. The result showed a significant target space overlap between the two medicines and this might give an insight into the mechanisms of the SWT formula in treating women’s disease as well as other indications.

### Validation using published literature

This study tried to show if a TOM formula can be used to treat the main indication as well as other indications and complications associated with the main indication (side effects) through target space analysis as well as by investigating the medicinal efficacy of individual herbs of a given TOM formula. To investigate the medicinal efficacy of individual herbs, we searched PubMed for scientific literature using herb names as a keyword. Prescription1-Obesity is a TOM formula that is known to be effective for Obesity. It is composed of seven herbs, Cyperus rotundus, Glycyrrhiza uralensis, Pinellia ternate, Triticum aestivum, Wolfiporia cocos, Atractylodes macrocephala, and Citrus reticulate. Cyperus Rotundus has been used as an anti**-**inflammatory, antioxidant, antidepressant, anti-obesity, and anti-diabetic[35–38]. Glycyrrhiza Uralensis has been also used as an anti-inflammatory and anti-obesity [39, 40]. Similarly, both Pinellia Ternate and Triticum Aestivum have been used as anti-obesity agents [41, 42]. Wolfiporia Cocos has been used as an antidepressant, anti-inflammatory, and anti-oxidant agent [43–45]. Atractylodes Macrocephala on the other hand has been used as an anti-inflammatory, anti-obesity, osteoporosis, and anti-gastrointestinal dysfunction [46–48]. Citrus reticulate has been used as an anti-inflammatory, anti-digestive, antifungal, and to prevent obesity and type 2 diabetes [49, 50]. Prescription2-Anxiety is a TOM formula that is known to treat Anxiety. It is composed of fourteen (14) herbs, Rehmannia glutinosa, Cyperus rotundus, Ziziphus jujube, Zingiber officinale, Pinellia ternate, Trichosanthes kirilowii, Glycyrrhiza uralensis, Ligusticum sinense, Coptis deltoidea, Foeniculum vulgare, Cullen corylifolium, Amomum villosum, Pachyma hoelen, and Phellodendron amurense. Rehmannia glutinosa is known to have anti-inflammatory [51, 52], immune stimulator effect [53], neuroprotective effect [54], and anti-fatigue [55] effects. Cyperus rotundus is known to have neuroprotective effect [56], central nervous effect [57], gastrointestinal effect [58], antidiabetic [59], and antiplatelet [60] effects. Ziziphus jujube is reported to have anxiolytic [61], constipation [62], hypotensive effect, and Immunostimulant effects [63]. Zingiber officinale is known to have anxiolytic effects[64], anti-diabetic and hypolipidaemic effects [65], and antioxidant and anti-inflammatory effects [66, 67]. Similarly, Pinellia ternate is reported to have anxiolytic, anti-inflammatory [68], anti-arrhythmic, lowering blood pressure, and anti-obesity effects [69]. Trichosanthes kirilowii is known for its anti-inflammatory and antioxidant activities [70, 71]. Glycyrrhiza uralensis and Ligusticum sinense are known to have anti-fatigue and anti-bacterial activities, respectively [72, 73]. Moreover, the earlier one is known for its role in moderating the characteristics of toxic herbs – detoxification [72]. Coptis deltoidea is reported to be effective for anti-inflammatory, antifungal, anti-bacteria, neuroprotection, gastrointestinal infections, type 2 diabetes and its cardiovascular complications [74–76]. Similarly, Foeniculum vulgare and Cullen corylifolium are reported to have anxiolytic activity, antidepressant, antimicrobial activity, anti-inflammatory, immunomodulatory, astringent, and estrogenic efficiency [77–81]. Prescription3-Diabetes is a TOM formula that is known to treat Diabetes Mellitus. This TOM formula is composed of ten herbs, Liriope muscari, Atractylodes macrocepha-la, Zingiber officinale, Schizandra Chinensis, Bos taurus domesticus, Panax ginseng, Anemarrhena asphodeloides, Coptis deltoidea, Rehmannia glutinosa, Nelumbo nucifera. Zingiber officinale is known to have an anti-diabetic effect, anti-inflammatory, antithrombotic, and anti-hypotensive effects [82, 83]. Schizandra Chinensis and Liriope muscari are known to have antioxidant, anti-inflammatory, hypoglycemic, antihypertensive, and anti-hyperglycemic effects [84–87]. Both Panax ginseng and Anemarrhena asphodeloides are reported to have anti-diabetic effects, improve insulin sensitivity and attenuate the development of diabetes, hypoglycemic activity, and anti-fatigue effects [88–91]. Rehmannia glutinosa is also reported to have anti-diabetic, hypoglycemic, and Immuno-enhancement effects [51]. Nelumbo nucifera and Coptis deltoidea are known to have antifungal effects, anti-inflammatory effects, anti-obesity effects, antioxidant effects, and hypoglycemic activities [92–94]. Prescription4-Hypertension is a TOM formula that is known to treat Hypertension. The formula is composed of 10 herbs, Angelica Sinensis, Cinnamomum aromaticum, Astragalus membranaceus, Astragalus complanatus, Tribulus terestris, Rehmannia glutinosa, Morinda Officinalis, Paeonia lactiflora, Uncaria rhynchophylla, and Epimediumbrevicornum. The first eight herbs are known to have antihypertensive, antioxidant, anti-inflammatory, alleviate constipation and intestinal inflammation, and anti-fatigue effects [95–99]. Uncaria rhynchophylla is reported to have anxiolytic, treat insomnia, and antidepressant effects [100, 101]. Lastly, Epimedium brevicornum is associated with estrogen biosynthesis promotion [102].

## Conclusions

TOM formula usually employs multi-component multi-target therapeutics, unlike most pharmaceutical drugs which are designed with a single active component or ingredient that targets a specific ion channel, receptor, regulatory protein, or enzyme that caused a disease. In this study, we employed target space analysis to provide scientific evidence about the efficacy of TOMs by investigating the target spaces of TOM formula and small-molecule drugs, identifying the overlapped targets between the two medications, and analyzing the potential of TOM formula to treat the main indication with side effects. To verify our results, we analyzed the SWT formula, a popular TCM formula, composed of four herbs and commonly used to treat various women’s diseases. The result of target space analysis between SWT formula and small-molecule drugs known to treat women’s disease were consistent with our findings. In conclusion, this study provides scientific support for the efficacy of TOMs and we believe our study will have a positive contribution to further the study of the mechanisms of action of TOMs.

## Materials and methods

### Data curation and preprocessing

The most important task in traditional medicine, such as herbal medicine, research is the successful annotation of herbal ingredients in chemical space. Several herbal databases are built up to date, such as the TCM Database@Taiwan [103], the Traditional Chinese Medicine Database (TCMD) [104], and Compound Combination-Oriented Natural Product Database with Unified Terminology (COCONUT) [105]. In this study, COCONUT was mainly used since it integrated a large size of herbal medicine formula and targets from various publicly available databases and published literature. Furthermore, we used TOM formulas and small molecule drugs that are known to treat Anxiety, Diabetes mellitus, Epilepsy, Hypertension, Obesity, and Schizophrenia.

**Fig. 7:**
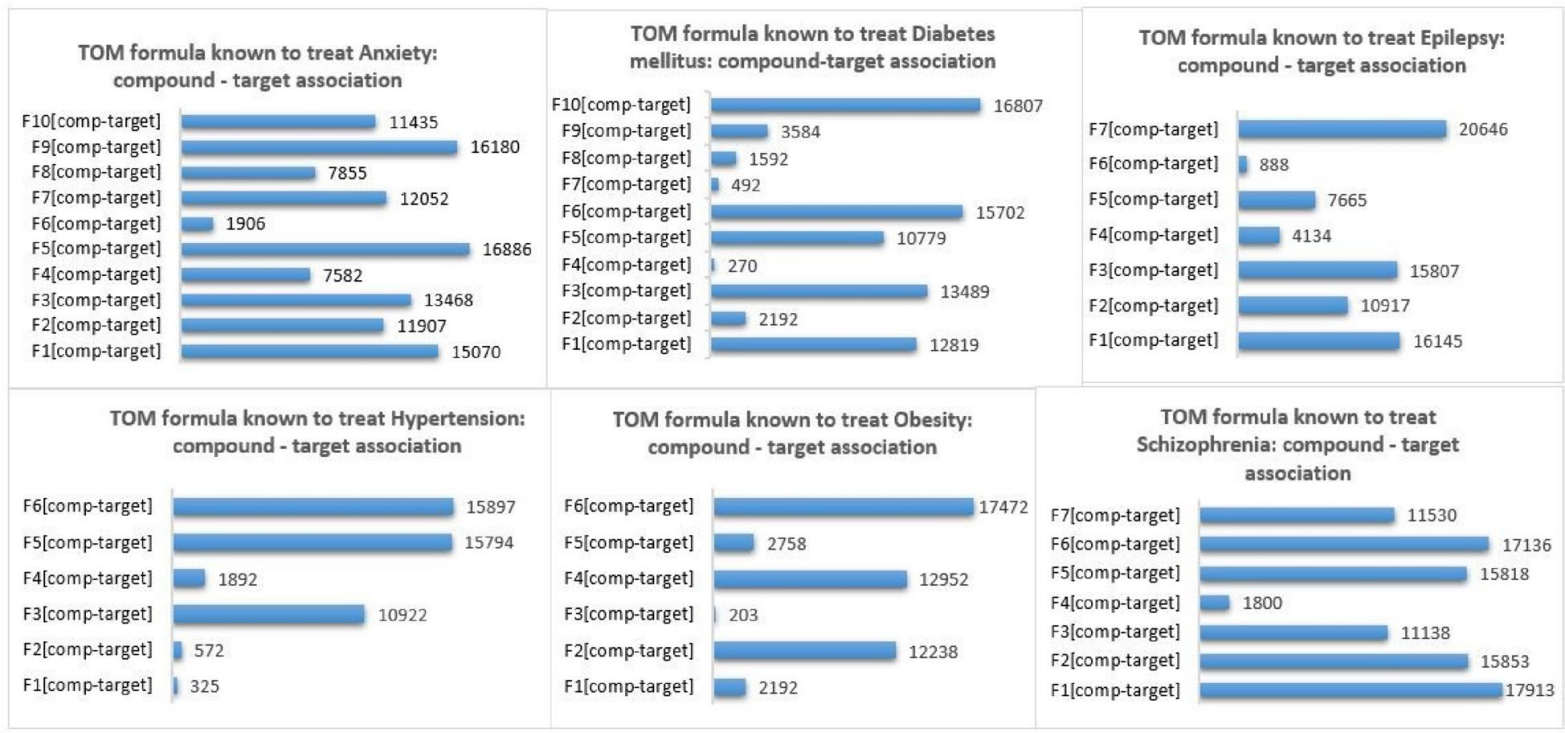
Traditional oriental medicine compound – target association dataset. Compound-target associations for 10 TOM formulas known to treat Anxiety, 10 TOM formulas known to treat Diabetes mellitus, 7 TOM formulas known to treat Epilepsy, 6 TOM formulas known to treat Hypertension, 6 TOM formulas known to treat Obesity, 7 TOM formulas known to treat Schizophrenia

The overall workflow and scheme of this study are shown in Fig. 2. The traditional herbal medicine dataset used for this work contains TOM formula–phenotype, TOM formula–herb, herbal ingredient–phenotype, and herbal ingredient–target associations. On the other hand, small-molecule drug-related data, such as small-molecule drug–target association, efficacy, and side effects were collected from DrugBank [106], SIDER [23, 24], and published literature. Table 6 and Fig. 7 showed statistics of TOM and small-molecule drug-related data used in this study.

**Table 6:** Materials used in this study

Phenotype	Number of TOM formulas	Number of small-molecule drugs	Number of small-molecule drug-target association
Anxiety	10	6	64
Diabetes mellitus	10	41	207
Epilepsy	7	18	148
Hypertension	6	61	384
Obesity	6	16	62
Schizophrenia	7	31	349

ADMET properties (absorption, distribution, metabolism, excretion, and toxicity) play important role in drug development and discovery. This work used admetSAR [107] and BeautifulSoup python library to predict ADMET-related properties for the herbal ingredients and retrieve predicted ADMET-related properties, respectively. We filtered out potentially toxic herbal ingredients from this study using ADMET properties AMES toxicity, Carcinogenicity, and hERG inhibition with predicted value toxic, danger, and an inhibitor/strong inhibitor, respectively.

### Manual curation of small-molecule drugs and building Drug Module 1 and Drug Module 2 sets

Small-molecule drug-related data was collected from multiple resources: (i) small-molecule drugs and their respected targets were collected from DrugBank; (ii) side effects of small-molecule drugs were collected from DrugBank, SIDER, and published literature. Next, we built Drug Module 1 and Drug Module 2 – Drug Module 1 is a set of small-molecule drugs that are known to treat the main indication whereas Drug Module 2 is a set of small-molecule drugs that are known to treat the side effects of Drug Module 1. For instance, in the present study, we performed target space analysis between TOM formula and small-molecule drugs in which both are known to treat Obesity. Here, Obesity is the main indication and a set of small-molecule drugs that are known to treat Obesity are classified as Drug Module 1. However, Small-molecule drugs (Drug Module 1) that are used to treat Obesity are known to cause side effects such as Insomnia, Tremor, Hypertension, Constipation, and Fatigue. Next, we built Drug Module 2- a set of small-molecule drugs – that are known to treat the side effects of Drug Module 1. Therefore, the target space analysis is designed to find out if a single TOM formula can be used to treat the main indication (Obesity) and the side effects (Insomnia, Tremor, Hypertension, Constipation, and Fatigue).

### Target space design and statistical model

The purpose of the target space analysis was to provide scientific evidence about the efficacy of herbal medicines. In the target space analysis, we identified the targets overlapped between TOM formula and small-molecule drug target spaces in which both medicines are known to treat the same disease. Moreover, previous studies have reported that small-molecule drugs are known to have various effects in direct administration such as undesired effects and it is a common practice to use additional small-molecule drugs to treat these side effects. Therefore, this study tried to investigate if a TOM formula could be used to treat the main indication as well as other indications (side effects) through TOM formula target space analysis and by investigating the medicinal efficacy of individual herbs. For instance, TOM formula A, composed of five herbs, is designed to treat Diabetes mellitus (main indication). The hypothesis we want to prove in this study is the effectiveness of TOM formula A to treat both the main indication (Diabetes mellitus) and potential side effects; can TOM formula A (herb1, herb2, herb3, herb4, herb5) treat the main indication with side effects? This work will be of help for traditional medicine-related studies by providing scientific evidence about the efficacy of TOMs as well as the holistic nature of herbal medicines. We used support to measure the significance of the target space overlap between the two target spaces, the TOM formula target space, and the small-molecule drug target space (Table 7). Furthermore, as an application of our method, we evaluated our work using a popular Traditional Chinese medicine formula called Si-Wu-Tang (SWT) Formula.

$$Support \left(X\to Y\right)=\frac{Support\left(X\cap Y\right)}{Support\left(X\right)}$$

where X- Drug target space, Y- Tom formula target space

**Table 7:** Overview of target space analysis

	Target A	Target B	Target C	Target D	Target E	Target n	Support
TOM formula 1	1	0	1	1	0	1	S1
TOM formula 2	1	1	0	0	1	0	S2
TOM formula 3	0	0	1	1	0	1	S3
……	……	……	……	……	……	……	……
TOM formula n	1	1	0	0	1	1	Sn

TOM Formula 1 to TOM Formula n are known to treat disease X, and Target A to Target n are the targets of pharmaceutical drugs known to treat disease X. In the target space analysis, we analyze TOM formula compounds if they are associated with these pharmaceutical drug targets and measure the significance of target overlap using Support

In this study, we used a weighted average, also known as a weighted mean, to calculate the overall score of a single TOM formula. As we can understand from its name, a weighted average differs from a simple arithmetic average since each value will be assigned different weights according to the importance each value carries. In the current study, the scores associated with the main indication are considered more significant as a specific formula is designed to treat the main indication and therefore it is given higher weight.

$${\text{W}}= {\sum }_{{\text{i}}=1}^{{\text{n}}}{{\text{w}}}_{{\text{i}}}{{\text{X}}}_{{\text{i}}}$$

where W = weighted average; n = number of values to be averaged; w_i_ = weight value; X_i_ = values to be averaged; ${\Sigma }_{{\text{i}}=1}^{{\text{n}}}{{\text{w}}}_{{\text{i}}}=1.$

The support values of target overlap between TOM formula A and small-molecule drugs (Drug Module 1) in which both are known to treat the same disease, disease X, were given 50% weight. On the other hand, the remaining 50% weight was equally distributed for the support values of target space overlap between TOM formula A and small-molecule drugs (Drug Module 2), which are known to treat the side effects of Drug Module 1. The weighted average value indicates the score a single traditional oriental medicine formula has to treat both the intended indication and other indications – side effects. However, the highest weighted average score does not imply that the reported TOM formula is ready to be used by human patients. The present study recommends further study and in-depth experiment to be carried away on those TOM formulas with the highest weighted average score to treat both the intended and unintended indications.

### Supplementary Information

**Additional file 1.** Shows the target space analysis result between the TOM formula and small-molecule drugs. In the current study, we analyzed 10 formulas known to treat Anxiety, 10 formulas known to treat Diabetes mellitus, 7 formulas known to treat Epilepsy, 6 formulas known to treat Hypertension, 6 formulas known to treat Obesity, and 7 formulas known to treat Schizophrenia.

**Additional file 2.** Shows SWT formula-target association. We provided SWT formula-herb-compound-target associations.

**Additional file 3.** Shows Drug Module 1-target associations. Drug Module 1 is a set of small-molecule drugs known to treat Women’s diseases such as climacteric syndrome, menstrual discomfort, and other estrogen-related diseases.

**Additional file 4.** Shows Drug Module 2-target associations. Drug Module 2 is a set of small-molecule drugs known to treat the side effects of Drug Module 1. All the additional files (Additional file 1, Additional file 2, Additional file 3, and Additional file 4) are available in the GitHub repository (https://github.com/BSRC-Resource/Target-Space-Analysis).

## Data Availability

The datasets analyzed during the current study are available in the GitHub repository (https://github.com/BSRC-Resource/Target-Space-Analysis). Additional file 1 (Target space analysis of TOM formula), Additional file 2 (SWT formula-target association), Additional file 3 (Drug Module 1-target association), and Additional file 4 (Drug Module 2-target association), are available in the BSRC-Resource/Target-Space-Analysis GitHub repository.

## Abbreviations

ADMET: Absorption, distribution, metabolism, excretion, and toxicity
COCONUT: Compound Combination-Oriented Natural Product Database with Unified Terminology
Drug Module 1: A set of small-molecule drugs known to treat disease X
Drug Module 2: A set of small-molecule drugs known to treat the side effects of Drug Module 1
Phen A: Phenotype A
Phen B: Phenotype B
SWT: Si-Wu-Tang
TCM: Traditional Chinese medicine
TCMD: Traditional Chinese Medicine Database
TOM: Traditional Oriental Medicine
